# Regulation of p63 Protein Stability via Ubiquitin-Proteasome Pathway

**DOI:** 10.1155/2014/175721

**Published:** 2014-04-15

**Authors:** Chenghua Li, Zhi-Xiong Xiao

**Affiliations:** Center of Growth, Metabolism and Aging, Key Laboratory of Biological Resources and Ecological Environment of Ministry of Education, College of Life Sciences, Sichuan University, Chengdu 610064, China

## Abstract

The p53-related p63 gene encodes multiple protein isoforms, which are involved in a variety of biological activities. p63 protein stability is mainly regulated by the ubiquitin-dependent proteasomal degradation pathway. Several ubiquitin E3 ligases have been identified and some protein kinases as well as other kinds of proteins are involved in regulation of p63 protein stability. These regulators are responsive to diverse extracellular signaling, resulting in changes of the p63 protein levels and impacting different biological processes.

## 1. Introduction

The p53 family member, p63 gene, is located on human chromosome 3q27–29. In contrast to the high frequency of p53 mutations in cancers, p63 gene is rarely mutated [1, 2]. However, up to 60% of squamous cell carcinomas show elevated p63 protein levels [3]. In addition, mutations in the p63 gene have been linked to several human developmental diseases. A vast body of evidence demonstrates that p63 are key transcription factors involved in cell growth, proliferation, apoptosis, and differentiation and play an essential role in epithelial stem cell biology and development [4–8]. Due to their key roles in a variety of essential biological processes, abundances of p63 proteins are tightly controlled. Ubiquitin-dependent proteasomal degradation is the most important way to eliminate cellular p63 proteins. Some important regulators, including ubiquitin E3 ligases, kinases, and proteins in other classes, have been reported to control p63 degradation. Multiple extracellular signalings, such as growth factor signaling and genotoxic stress, impact these regulators, which in turn modulate protein stability of p63 [9, 10]. This review is aimed at understanding the molecular mechanisms, by which p63 protein stability is regulated, and the players in modulating ubiquitin-dependent proteasomal degradation of p63 proteins.

## 2. Isoforms of p63 and Their Biological Functions

**Figure 1:**
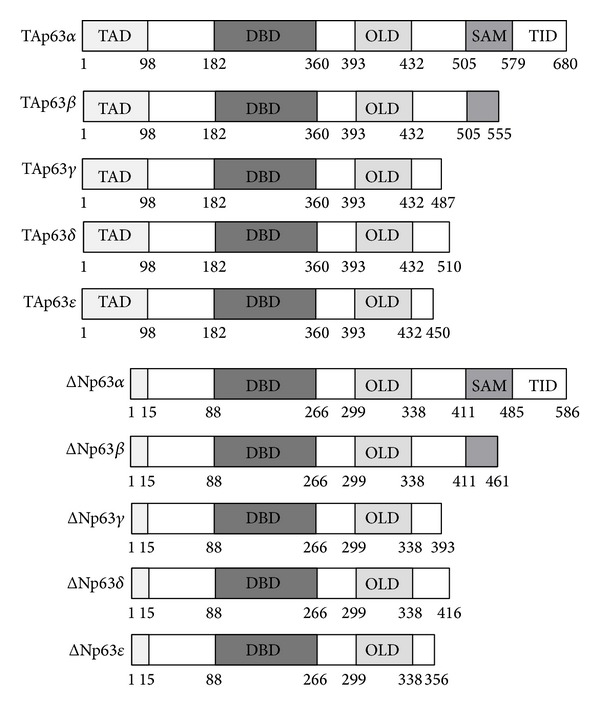
Schematic presentation of p63 isoforms. TAD: transactivation domain; DBD: DNA-binding domain; OLD: oligomerization domain; SAM: sterile alpha motif; TID: transinhibitory domain.

The p63 gene consists of 15 exons that can be transcribed from two transcriptional start sites. The transcript from 5′ promoter of p63 gene proceeding to the first exon encodes TA isotypes of p63 proteins with the full transactivation domain (TAD) homologues to that of p53 on the N-terminus, while transcript from the cryptic 3′ intronic promoter gives rise to ΔN isoforms containing a different and weaker domain capable of transactivation. Both TA and ΔN isotypes can undergo alternative splicing to generate different carboxy-termini, including *α*, *β*, *γ*, *δ*, and *ε*. Thus, p63 gene can encode at least 10 different p63 isoforms, TA(*α* ~ *ε*) and ΔN(*α* ~ *ε*) (as depicted in Figure 1). Each p63 isoform possesses a DNA-binding domain (DBD) and an oligomerization domain (OLD), both of which are highly homologous to those of p53 protein. Among these p63 isoforms, p63*α* contains a full-length C-terminus consisting of a sterile alpha motif (SAM) for protein interaction and a transinhibitory domain (TID), whereas other isoforms have truncated C-termini due to alternative splicing [11–13]. Evidence from human genetics and animal models reveals that p63 proteins play crucial roles in stratification of squamous epithelia, differentiation of mature keratinocytes, and epidermal morphogenesis during development [14, 15]. Multiple p63 isotypes (both TA and ΔN isoforms) are expressed in keratinocytes and they are differentially modulated during differentiation [16, 17].

Endogenous TAp63 proteins are barely detectable in embryos and adult (except in oocytes), presumably because of their low expression or rapid degradation as well as lack of antibodies able to detect weak expression [18]. It is well supported that, like p53, TAp63 proteins promote cell cycle arrest and apoptotic cell death via activating proapoptotic targets, such as Puma, Bax, and Noxa in somatic cells [1, 19, 20]. In oocytes, TAp63*α* expresses at relatively higher levels and functions as a quality control factor in the female germline, upon genotoxic stress [21–23]. TAp63 knockout mice are highly tumor prone and develop metastatic disease, reaffirming the antitumor activities of TAp63 [5, 6]. Loss of TAp63 also results in premature aging and reduced lifespan in mice [5, 24]. Recently, increased obesity, insulin resistance, and glucose intolerance were reported in TAp63-null mice [25].

ΔNp63, especially ΔNp63*α*, are predominant p63 isoforms, which are overexpressed in a highly specific manner in the embryonic ectoderm and the basal regenerative compartment of epithelial tissues, such as skin, teeth, and hair [26]. Similar to mice deleted with all p63 isoforms, ΔNp63-null mice have striking developmental defects including truncated forelimbs, the absence of hind limbs, and a lack of stratified epidermis [27]. In contrast to the strong transactivation function of TAp63 proteins, ΔNp63 isoforms were traditionally believed to inhibit p53 members including p53, TAp63, and TAp73 proteins via forming complexes with them or competitive binding to p53-responsive elements. This transcriptional repressor activity enables ΔNp63*α* to promote cell proliferation and tumorigenesis under certain circumstances [4, 11, 19, 28, 29]. According to this model, the fine balance between the TA and ΔN isotypes determines the function of p63 proteins. However, mounting evidence reveals that ΔNp63 has an intrinsic transcriptional activity owing to a second TA domain (TA2). ΔNp63*α* has been shown to regulate the expression of several adhesion molecules, including integrins (*β*1, *β*4, and *α*6) and desmosome protein PERP, as well as MAP kinase phosphatase 3 (MKP3), heat shock protein 70 (HSP70), multidrug resistant gene 1 (MDR-1), and ATM kinase, implicating its functions in cell growth, invasion, survival, drug resistance, and DNA repair [8, 10, 30–32].

## 3. Properties of p63 Protein Stability

Due to their potent proapoptotic activities, TAp63 proteins are generally expressed at very low levels, likely owing to the transcriptional regulation. Alternatively, TAp63 proteins are highly labile. It has been documented that the half-life of TAp63*γ* is about 1.5 hours, and an unknown factor may play as a feedback regulator of TAp63 degradation. ΔNp63 proteins are much more stable than TAp63 [33]. They are found overexpressed in keratinocyte and squamous carcinoma cells and associated with proliferation. It has been shown that while ΔNp63 undergoes degradation [34], TAp63 accumulates in response to some extrinsic stresses such as actinomycin D, bleomycin etoposide, and UV irradiation [10]. Some intrinsic physiological processes such as cell differentiation are also likely to regulate degradation of p63 proteins [16, 35, 36].

Although lysosomal degradation may be involved in regulation of p63 abundance, the most important way of p63 degradation is the proteasome-dependent pathway [10]. The stabilities of p63 proteins are modulated by diverse posttranslational modification, such as phosphorylation, ubiquitylation, SUMOylation, and ISGylation, in which various proteins are involved [26, 37–39].

## 4. E3 Ligases Targeting p63 for Proteasomal-Mediated Degradation

As the primary pathway of p63 protein degradation, ubiquitin-dependent proteasomal degradation of p63 proteins was reported by several laboratories. E3 ligase-mediated ubiquitylation is the essential step for proteasomal degradation of a specific protein. Up to now, several E3 ligases for p63 proteins have been identified (as listed in Table 1).

### 4.1. Nedd4

Nedd4 is the first identified E3 ligase for p63. Using a yeast-two-hybrid screening system, Bakkers et al. found that two modifying enzymes, the E3 ubiquitin ligase Nedd4 and the SUMO-conjugating enzyme Ubc9, bind to distinct sites in the unique C-terminal region of ΔNp63*α*. These physical interactions lead to ubiquitylation and SUMOylation of ΔNp63*α*, resulting in vulnerability of ΔNp63*α* to proteasomal degradation [36]. In zebrafish embryos, ΔNp63*α* are expressed at a high level on the dorsal side, due to the restricted expression of* ubc9.1* and* nedd4* in this region [36]. However, how does the Ubc9-mediated SUMOylation of Lys582 destabilize ΔNp63*α* remains unknown. In addition, whether TAp63*α*, which shares the same C-terminus with ΔNp63*α*, is modulated by Nedd4 and Ubc9 remains to be investigated. It was recently reported that ΔNp63*α* can transcriptionally repress Nedd4 [40], suggesting that downregulation of Nedd4 may function as a feedforward pathway to increase ΔNp63*α* intracellular concentration under certain circumstances.

### 4.2. Itch

Itch is Nedd4-like ubiquitin E3 ligase found to target p63 for ubiquitin-mediated proteasomal degradation. Work from Melino's group found that Itch directly binds to the PPPY motif existing in the SAM domain of p63*α*. This physical interaction leads to ubiquitylation of either TA- or ΔN-p63*α* isoforms, consequently promoting their proteasomal degradation. A Y→F substitution in the PPPY motif can abolish the binding of Itch and significantly increases p63*α* protein stability. Their data suggest that Itch plays a fundamental role in controlling endogenous p63 protein levels and regulating p63-mediated physiological functions, particularly in the epidermis and keratinocytes [26]. This is similar to the case of p73, which is also ubiquitylated and targeted to degradation by Itch through PPPY-Itch interaction [41]. And this is also consistent with cases of p63 degradation mediated by Nedd4 and WWP1, which both are analogues of Itch and bind to PPPY motif of p63 [36, 42].

Nevertheless, Calabro group found that Itch bound to a different region of p63, the region encompassing aminoacids 109 to 120 of TAp63 (corresponding to amino acids 15 to 26 of ΔNp63*α*). This association between Itch and N-termini of p63 promotes degradation of all p63 isoforms, including both TA- and ΔN-isotypes [37].

### 4.3. WWP1

Li et al. found that WWP1, the homologue of Itch, can also bind to the PPPY motif of either TAp63*α* or ΔNp63*α* and ubiquitinate them in cultured mammalian cells, consequently promoting their proteasomal degradation. Additionally, WWP1 can target endogenous ΔNp63*α* proteins for degradation and sensitizes immortalized breast epithelial cells to chemotherapeutic drug doxorubicin-induced apoptosis. Intriguingly, WWP1 can be upregulated at both mRNA and protein levels upon chemotherapeutic drug treatment in a p53-dependent manner [42]. This suggests that ΔNp63*α*, which is well documented to confer cells to resistance to DNA damage agent-mediated apoptosis [43], may be destabilized by accumulated WWP1 E3 ligase, resulting in cell death, under genotoxic stress.

### 4.4. Fbw7

Although several groups reported that MDM2, which is a nuclear E3 ubiquitin ligase playing key roles in controlling cellular p53 abundance, cannot individually mediate ubiquitylation of p63 [44–46], Galli et al. found that MDM2 can facilitate ubiquitylation of p63 mediated by another E3 ligase, Fbw7. Upon DNA damage or keratinocytes differentiation, MDM2 binds to the C-terminal SAM region of ΔNp63*α* in the nucleus and promotes its translocation to the cytoplasm; then p63 is targeted for degradation by the Fbw7 E3 ubiquitin ligase in the cytosome. In this process, GSK3 kinase activity is required for efficient degradation of p63 by Fbw7 [35].

### 4.5. Pirh2

Pirh2 (p53-induced RING-H2) is another E3 ligase found to target p63 for degradation. It was recently reported that Pirh2 physically interacts with TAp63 and ΔNp63 and targets them for polyubiquitylation and subsequently proteasomal degradation. This Pirh2-mediated posttranscriptional regulation of p63 may modulate keratinocytes differentiation [47]. Arsenic trioxide, a frontline agent for acute promyelocytic leukemia, stimulates Pirh2 expression and consequently promotes proteasomal degradation of ΔNp63*α* but not TAp63*α* [48]. This is interesting and useful, because ΔNp63*α* is generally considered as a potent oncoprotein playing key roles in tumor cell proliferation/survival, whereas TAp63*α* is a tumor suppressor with proapoptotic activity. It remains obscure why these two isoforms, which can both be targeted for degradation by Pirh2, have this discrepancy in response to arsenic trioxide treatment.

## 5. Kinases Involved in p63 Protein Degradation

Phosphorylation mediated by diverse kinases plays key roles in protein degradation. So far, several kinases have been reported to be involved in p63 protein stability (as listed in Table 1).

### 5.1. ATM, CDK2, and p70s6K

Sidransky group found that kinases including ATM, CDK2, and p70s6K can phosphorylate ΔNp63*α* in HNSCC (head and neck squamous cell carcinoma) cells upon DNA damage. Phosphorylation at S385, T397, and S466 mediated by these kinases, respectively, promotes degradation of ΔNp63*α* [49]. Given that ΔNp63*α* can transcriptionally regulate ATM [50], the latter may function as a feedback regulator via a p53/MDM2-like loop to tightly control protein level of ΔNp63*α*.

### 5.2. IKK**β**

TAp63*γ* exhibits potent proapoptotic activity due to possessing a full-length transactivation domain and lacking a transinhibitory domain. Normally, TAp63*γ*  expresses at very low levels in cells. It was reported that, in response to *γ* radiation or tumor necrosis factor-*α* (TNF-*α*), I*κ*B kinase *β* (IKK*β*) is activated and phosphorylates TAp63*γ* (but not ΔNp63*γ*) at its N-terminus; this phosphorylation can significantly block ubiquitylation and possible degradation of TAp63*γ* in H1299 and HEK293 cells, resulting in elevated protein levels of cellular TAp63*γ* [33, 51].

On the contrary, Sidransky group found that cytokine- or chemotherapy-induced stimulation of IKK*β* kinase promotes ubiquitin-mediated proteasomal degradation of ΔNp63*α* in the human head and neck cancer cell line JHU-022, consequently augmenting transactivation of p53 family-induced genes involved in the cellular response to DNA damage [52]. It is unclear how IKK*β* kinase exhibits opposite effects on stabilities of these two different isoforms. An interpretation is that ΔNp63*α* and TAp63*γ* possess different C-terminus or N-terminus.

### 5.3. HIPK2

Homeodomain-interacting protein kinase 2 (HIPK2) is an evolutionarily conserved serine/threonine kinase involved in the regulation of gene transcription during development and in cell response to several types of stress. HIPK2 is often activated under diverse genotoxic stimuli, including treatment with ultraviolet, ionizing irradiation, and anticancer drugs such as cisplatin, doxorubicin, and roscovitine [53, 54]. Lazzari et al. found that HIPK2 phosphorylates ΔNp63*α* at T397 residue in response to chemotherapy. This modification promotes proteasomal degradation of ΔNp63*α*, which is mediated by neither Itch nor MDM2 [55].

### 5.4. c-Abl

c-Abl (also known as ABL1) is a tyrosine kinase, which can be activated upon DNA damage [56, 57]. Gonfloni et al. recently reported that, in response to cisplatin treatment, c-Abl phosphorylates TAp63*α* on Y149, Y171, and Y289 residues, resulting in increased protein stability of TAp63*α*. This can lead to accumulated TAp63*α* proteins and eventually cell death in mouse oocytes [22].

Another group found that c-Abl phosphorylates Y55, Y137, and Y308 residues of ΔNp63*α* and stabilizes it via inducing its binding to Yes-associated protein (YAP), consequently regulating cell viability in head and neck cancer cells [58]. Since Y149, Y171, and Y289 residues of TAp63*α* are different from Y55, Y137, and Y308 of ΔNp63*α*, it is interesting why TAp63*α* and ΔNp63*α* are stabilized by c-Abl in different mechanism. It also remains unclear if other isoforms of p63 can be regulated by c-Abl, given that these six putative c-Abl phosphorylation sites are included in all of p63 isoforms.

### 5.5. Other Kinases

In addition to the abovementioned kinases, Plk1 [59], p38 [60], GSK3 [35], and Raf1 [16] kinases may be also involved in proteasome-dependent degradation of p63.

## 6. Other Proteins Regulating p63 Protein Stability

Besides E3 ubiquitin ligases and kinases, several other proteins were found to be related to protein stability of p63 (as listed in Table 1). Most of them function as regulators or cofactors of the abovementioned E3 ligases or kinases to promote or inhibit proteasomal degradation of p63.

### 6.1. p53

Ratovitski group found that p53 can bind to DNA-binding domain of p63 in the absence of DNA and promote p63 degradation through a pathway mediated by caspase-1. Interestingly, this p63 caspase-mediated degradation of p63 resulting from p53-p63 physical interaction needs no further p63 posttranslational modifications [29].

### 6.2. Stratifin, RACK1, and Stxbp4

In another work, Ratovitski et al. found that, in response to DNA damage, stratifin binds to the putatively phosphorylated ΔNp63*α* protein and regulates its level through nuclear-cytoplasmic trafficking, while scaffold protein RACK1 (receptor for activated C kinase 1) subsequently targets ΔNp63*α* into a 26S-dependent proteasomal degradation pathway. Though overexpression of RACK1 dramatically enhances ubiquitylation of ΔNp63*α*, it needs further confirmation if RACK1 serves as an E3 ligase in this pathway [61].

Since it has been documented that RACK1 can regulate FAK activity [62], it is also possible that RACK1 promotes ubiquitylation via affecting phosphorylation of p63. Notably, it has been reported that stratifin is transcriptionally regulated by p63 and p53 [28, 63]. So this modulation of p63 mediated by stratifin/RACK1 may also form a regulation loop.

Another scaffold protein Stxbp4 was found to counteract this RACK1-dependent degradation pathway to maintain high basal levels of ΔNp63 in stratified epithelial cells under normal growth conditions; but in response to DNA damage, Stxbp4 is downregulated, correlating with ΔNp63*α* destabilization mediated in part by RACK1 [64].

### 6.3. Other Proteins

Hildesheim et al. reported that under genotoxic stress Gadd45a mediates activation of p38 MAP kinase and consequently downregulates ΔNp63*α* protein [60]. Wang et al. found that Cables1 (CDK5 and Abl enzyme substrate 1) associates with both TAD and SAM regions of TAp63*α* to protect TAp63*α* from proteasomal degradation [65]. Bernassola et al. found that the promyelocytic leukaemia protein (PML) physically interacts with TAp63*α* and ΔNp63*α* and increases their protein levels [66]. According to results of Di Costanzo et al., homeodomain protein Dlx3 physically interacts with and activates Raf1 kinase, resulting in enhanced phosphorylation and degradation of ΔNp63*α* [16]. As mentioned above, YAP and Ubc9 are also involved in regulation of p63 degradation [36, 58]. Recently, we found that peptidyl-prolyl isomerase Pin1 inhibits binding of WWP1 E3 ligase to p63*α*, consequently preventing its proteasomal degradation [67].

## 7. Summary and Prospects

Taken together, current studies reveal that p63 protein stability is tightly controlled and closely correlated with cell proliferation, cell death, and cell differentiation. Various signalings and stresses can affect p63 protein stability via regulating E3 ligases or activities of diverse kinases. Notably, the same residues or same regions are targeted by different E3 ligases or different signaling. For instance, T397 of ΔNp63*α* can be phosphorylated by several kinases, including CDK2, HIPK2, and Raf1, consequently promoting degradation of ΔNp63*α*; three E3 ligase homologues, NEDD4, Itch, and WWP1, target p63 for proteasomal degradation via binding to PPPY motif of p63*α* and p63*β*. Furthermore, the regulation of p63 protein stability is complex, since the effects of E3 ligases on p63 degradation are modulated by kinases and other regulators. Some of the factors can be transcriptionally regulated by p63, forming some regulation loops. Further investigations are required to clarify the relationships between different regulators in p63 protein stability upon exogenous signaling. Moreover, it is necessary to elucidate how different p63 isotypes are targeted to degradation during cell differentiation, since a switch in expression of different p63 isoforms is pivotal in this process [68].

On the other hand, it would be important to investigate key regulators for p63 degradation that are responsible for p63-related human developmental diseases. Like the paradigm of promising anticancer drug Nutlin-3, which can stabilize p53 via disrupting its association with MDM2 [69], it would be interesting to develop potent inhibitors targeting E3 ligases in order to stabilize TAp63 tumor suppressors or to destabilize ΔNp63 oncoproteins.

## Figures and Tables

**Table 1:** Regulators of p63 protein stability. +: positively regulates p63 protein stability; −: negatively regulates p63 protein stability. ∗: MDM2 functions as not an E3 ligase here, but a helper to promote translocation of ΔNp63*α* to cytoplasm [35].

Regulators	Stability regulation	p63 isoform	Interaction or modification sites	References
E3 ligases				
Nedd4	−	ΔNp63*α*	PPPY motif in SAM domain	[36]
Itch	−	ΔNp63*α*	PPPY motif in SAM domain	[26]
	−	All p63 isoforms	A region encompassing aminoacids 109 to 120 of TAp63 (aminoacids 15 to 26 of ΔNp63)	[37]
WWP1	−	p63*α*	PPPY motif in SAM domain	[42]
Fbw7	−	ΔNp63*α*	Likely a region encompassing S383 of ΔNp63*α*	[35]
Pirh2	−	TAp63 and ΔNp63		[47, 48]
Kinase				
ATM	−	ΔNp63*α*	S385 of ΔNp63*α*	[49]
CDK2	−	ΔNp63*α*	T397 of ΔNp63*α*	[49]
p70s6K	−	ΔNp63*α*	S466 of ΔNp63*α*	[49]
IKK*β*	+	TAp63*γ*	TAD of TAp63*γ*	[51]
	−	ΔNp63*α*	Unknown	[52]]
HIPK2	−	ΔNp63*α*	T397 of TAp63*γ*	[55]
c-Abl	+	TAp63	Y149, Y171, and Y289 of TAp63	[22]
	−	ΔNp63*α*	Y55, Y137, and Y308 of ΔNp63*α*	[58]
Plk1	−	TAp63	S52 of TAp63	[59]
p38	−	ΔNp63*α*	Unknown	[60]
GSK3	−	ΔNp63*α*	S383 of ΔNp63*α*	[35]
Raf1	−	ΔNp63*α*	S383 or T397 of ΔNp63*α*	[16]
Others				
Gadd45a	−	ΔNp63*α*	No direct interaction	[60]
p53	−	ΔNp63*α*	DBD of ΔNp63*α*	[29]
MDM2*	−	ΔNp63*α*	SAM domain	[35]
Cabbles1	+	TAp63*α*	TAD and SAM of TAp63*α*	[65]
YAP	+	ΔNp63*α*	PPPY motif in SAM domain	[58]
Dlx3	−	ΔNp63*α*	No direct interaction	[16]
Stratifin	−	p63*α*	RQTISFP motif in TID of p63*α*	[61]
RACK1	−	p63*α*	C-ter of p63*α*	[61]
Stxbp4	+	ΔNp63*α*	PPPY motif in SAM domain	[64]
Ubc9	−	ΔNp63*α*	K582 of ΔNp63*α*	[36]
PML	+	p63*α*	Unknown	[66]
Pin1	+	p63*α*	PPPY motif in SAM domain	[67]
